# Sperm DNA Fragmentation: Unraveling Its Imperative Impact on Male Infertility Based on Recent Evidence

**DOI:** 10.3390/ijms251810167

**Published:** 2024-09-22

**Authors:** Sofoklis Stavros, Anastasios Potiris, Ermioni Molopodi, Despoina Mavrogianni, Athanasios Zikopoulos, Konstantinos Louis, Theodoros Karampitsakos, Eleni Nazou, Dimdos Sioutis, Chrysi Christodoulaki, Charikleia Skentou, Angeliki Gerede, Athanasios Zachariou, Panagiotis Christopoulos, Periklis Panagopoulos, Ekaterini Domali, Peter Drakakis

**Affiliations:** 1 Third Department of Obstetrics and Gynecology, University General Hospital “ATTIKON”, Medical School, National and Kapodistrian University of Athens, 12462 Athens, Greece; sfstavrou@med.uoa.gr (S.S.); thanzik92@gmail.com (A.Z.); kostaslouisss@gmail.com (K.L.); theokarampitsakos@hotmail.com (T.K.); elenaz@med.uoa.gr (E.N.); dsioutis@gmail.com (D.S.); perpanag@med.uoa.gr (P.P.); pdrakakis@med.uoa.gr (P.D.); 2 Medical School, National and Kapodistrian University of Athens, 11527 Athens, Greece; ermionimol@gmail.com; 3 First Department of Obstetrics and Gynecology, Alexandra Hospital, Medical School, National and Kapodistrian University of Athens, 11528 Athens, Greece; dmavrogianni@med.uoa.gr (D.M.); kdomali@yahoo.fr (E.D.); 4 Department of Obstetrics and Gynecology, Chania General Hospital “St. George”, 73300 Chania, Greece; christodoulakichr@hotmail.com; 5 Department of Obstetrics and Gynecology, Medical School of the University of Ioannina, 45110 Ioannina, Greece; haraskentou@uoi.gr; 6 Department of Obstetrics and Gynecology, Democritus University of Thrace, 69100 Campus, Greece; agerede@otenet.gr; 7 Department of Urology, School of Medicine, Ioannina University, 45110 Ioannina, Greece; zahariou@otenet.gr; 8 Second Department of Obstetrics and Gynecology, Aretaieion University Hospital, Medical School, National and Kapodistrian University of Athens, 11528 Athens, Greece; panchrist@med.uoa.gr

**Keywords:** male infertility, unexplained infertility, sperm DNA fragmentation, DNA fragmentation index, DFI, assisted reproduction techniques

## Abstract

Male factors may be present in up to 50–70% of infertile couples and the prevalence of male infertility accounts for 20–30% of infertility cases. Understanding the mechanisms and causes behind male infertility remains a challenge, but new diagnostic tools such as DNA fragmentation might aid in cases where the routine semen analysis is insufficient. DNA fragmentation, which refers to damages or breaks of the genetic material of the spermatozoa, is considered one of the main causes of male infertility due to impaired functional capability of sperm. The aim of the present narrative review is to investigate and enlighten the potential correlation between DNA fragmentation and male infertility parameters such as the seminal profile and the reproductive outcomes. Comprehensive research in PubMed/Medline and Scopus databases was conducted and 28 studies were included in the present review. Fourteen studies provided data regarding the impact of DNA fragmentation and seminal parameters and showed a correlation of significantly lower sperm count, lower concentration, motility, and abnormal morphology with an increased DNA fragmentation index (DFI). Similarly, 15 studies provided data regarding the impact of DFI on reproductive outcomes. Two studies showed higher aneuploidy rates with higher DFI values, and seven studies showed significantly lower pregnancy rates and live birth rates with higher DFI values. Ultimately, the studies included in this review highlight, collectively, the importance of measuring sperm DFI in the assessment of male infertility. Further studies are needed to explore the effectiveness of interventions aiming to reduce DFI levels.

## 1. Introduction

Infertility is defined as the inability to conceive after one year of regular unprotected sexual intercourse [1]. It is estimated that 10–15% of couples and around 50–80 million couples worldwide are affected. While about half of infertility cases are attributed to female factors, male factors contribute to 20–30%, and another 20–30% result from common causes affecting both partners [2,3]. Recent data suggest that male factors may be present in up to 50–70% of infertility cases, although this wide range may not reflect global prevalence due to different data collection methods and cultural influences [4,5].

Understanding the mechanisms behind male infertility remains a significant challenge [6]. Currently, semen analysis is still a laboratory technique considered the gold standard for attempting to identify and evaluate male infertility [7]. This technique is based on the principle that infertility can often be triggered by several factors that alter seminal parameters, including sperm motility, morphology, liquefaction time, seminal volume, sperm concentration, and sperm motility [8]. In 2021, the World Health Organization (WHO) revised the published guidance and referred to seminal fluid parameters as “useful parameters”, indicating the imperfect association between the parameters and actual male infertility [9]. The latter led to other semen evaluating tools to explore further the field of unexplained male infertility. These tests include the evaluation of anti-sperm antibodies, sperm hyperactivation, acrosomal reaction, penetration in the zona pellucida, and sperm DNA fragmentation [10].

DNA fragmentation refers to damages or breaks of the genetic material of the spermatozoa, and as mentioned before, routine semen analysis is unable to estimate it. These damages can be caused by either intrinsic factors such as increased oxidative stress and defective maturation or external factors such as previous chemotherapy, smoking, higher temperature in the scrotum, and endocrine-disrupting compounds [11,12]. DNA fragmentation is considered one of the main causes of male infertility due to impaired functional capability of sperm [13]. Its negative effect on fertility also extends to assisted reproduction because spermatozoa with impaired DNA are able to fertilize an oocyte and, consequently, the limited repair mechanisms in the embryo [14]. Hence, high DNA fragmentation has been associated with negative reproductive outcomes and failure to reach the blastocyst stage in embryos created either in vivo or in vitro [15,16]. However, regardless of the increasing literature on the impact of sperm DNA fragmentation measured by the DNA fragmentation index (DFI), major societies such as the European Society of Human Reproduction and Embryology (ESHRE), in their recent guidance, either oppose DFI testing or consider it in the context of research [17].

The aim of the present narrative review is to investigate and enlighten the potential correlation between DNA fragmentation and male infertility parameters such as the seminal profile and the reproductive outcomes.

## 2. Literature Research

Comprehensive literature research was conducted across two major databases: Pubmed/Medline (2013–2024) and Scopus (2013–2024). Our research was limited to the last decade due to the implementation of newer techniques in measuring the DNA fragmentation index. The search terms used included “male”, “infertility”, “DNA Fragmentation”, “DNA Fragmentation Index”, “DFI”, and “Assisted Reproduction” with the administration of Boolean operators (OR, AND) combined with those keywords either used as presented, separately, or in combination. Two authors (AP and EM) conducted the literature search and abstract selection independently. The content of full-text publications that were eligible was further assessed. A third reviewer, S.S., was responsible for making the final decision on a study if the study was selected by only one reviewer. Additionally, the “snowball literature searching method” was applied to identify further relevant sources from the reference lists of selected articles.

All original articles on research conducted in the last decade in humans and written in English, with the main subject of the investigation being the role of sperm DNA fragmentation in male fertility, were included in this review. Similarly, secondary studies, such as reviews, systematic reviews and meta-analyses, articles written in another language than English, studies conducted in animal models, and studies referring to the effect of specific treatments on DFI, were excluded.

Regarding the quality assessment and the risk of bias assessment of the included studies, a critical evaluation of each study’s sample (size of the sample), methodology (study design and compared groups), outcome presentation (clarity and relevance of reporting outcomes), and confounding factors (potential biases and different methods of estimating DNA fragmentation) was performed. This critical evaluation helped with the interpretation of each study’s outcomes and the better presentation of our results and discussion on the effect of DNA fragmentation on male infertility. A formal risk of bias and quality assessment was not performed due to the narrative nature of this review.

**Figure 1:**
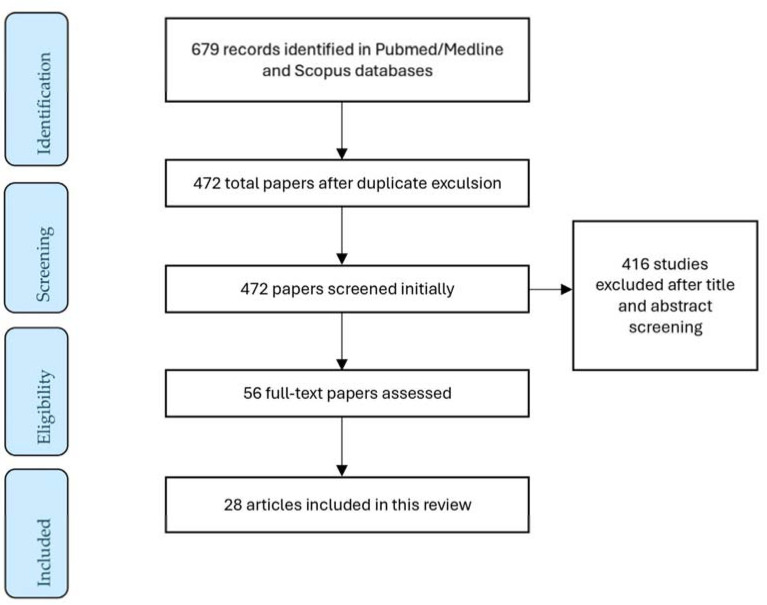
A flow diagram of the study selection process.

From the initial research, 679 articles were collected via PubMed/Medline and Scopus databases. A total of 472 were screened by title and abstract and 56 underwent full-text assessment. Ultimately, 28 articles were suitable for providing information in this literature review. The study selection process is depicted in Figure 1.

## 3. Impact of DFI on Seminal Parameters

Regarding the impact of sperm DNA fragmentation on seminal parameters, 14 studies have been identified. Two studies demonstrated no significant correlation between DNA fragmentation index values and conventional seminal parameters [18,19]. The remaining 12 studies showed at least one negatively affected seminal parameter. More specifically, six studies demonstrated a significant correlation between higher values of DNA fragmentation and lower values of lower seminal volume or concentration [20,21,22,23,24,25]. Zhang et al. showed significantly lower sperm concentration values, even for the study group with a DFI between 20% and 30% [24]. The same negative effect of DNA fragmentation has also been indicated by Green et al. at the threshold of 15% [23], showing that concentration and volume are among the first parameters to be affected, even at low DFI values.

Regarding motility, six studies showed a statistically significant association of higher DFI values with lower motility [21,22,23,26,27,28]. Lastly, six studies showed a significant association between DNA fragmentation and sperm morphology [20,25,27,29,30,31]. It is worth noting that teratozoospermia was the most statistically significant finding in the majority of the studies, with *p* values of 0.001. The sample and the main outcome of each included study are presented in Table 1.

## 4. Impact of DNA Fragmentation on Reproductive Outcomes

Regarding the impact of sperm DNA fragmentation on embryo kinetics, quality, and assisted reproduction outcomes, 15 studies have been identified. Five studies provide data regarding embryo kinetics and euploid status [32,33,34,35,36]. Only one study by Sun et al. showed no significant correlation between the DFI values and embryo aneuploidy [34]. It is worth mentioning that the cut-off selected by the authors was a DFI over and under 30% for group allocation. One study by Wdowiak et al. showed that lower DNA fragmentation was associated with faster embryo development to the blastocyst stage [36], and one study demonstrated that DFI values were inversely correlated with blastocyst viability status [35]. Lastly, two studies showed statistically significant higher aneuploidy rates with higher DNA fragmentation index values [32,33].

Five studies report data on fertilization rate as an outcome [32,34,37,38,39]. Interestingly, three of them report no significant difference in the fertilization rate among high- and low-DFI groups [34,37,38], and two studies report significantly lower fertilization rates [32,39]. It is worth mentioning that from the latter two studies, the study by Wang et al. demonstrated significantly lower fertilization rates only in the IVF comparison group and not in the ICSI group [32].

On the key clinical outcomes of pregnancy rate and live birth rate, our study included data from ten studies [10,32,34,37,39,40,41,42,43,44]. Three studies report no significant differences regarding the pregnancy rate [34,37,43]. However, in the study by Omrani et al., there was no reported pregnancy in the high-DFI group, and the result of no correlation between the DFI and pregnancy rate was based on the comparison of the control group with the moderate-DFI group [43]. The remaining seven studies report significantly higher pregnancy and live birth rate results with lower DFI values [10,32,39,40,41,42,44]. In the three studies with subgroups undergoing IVF and ICSI, a higher pregnancy rate was observed in the ICSI group for the same DFI values [41,42,44]. The sample and the main outcome of each included study in this section are presented in Table 2.

## 5. Discussion

The studies examined in the section on the impact of the DNA fragmentation index (DFI) on male infertility and sperm quality underscore the significance of sperm DNA fragmentation as a critical factor in male infertility. They also confirm the relationship between the DFI and the reduced likelihood of successful conception, higher rates of miscarriage, and embryo development issues. Furthermore, they reveal that advanced age and human habits such as smoking and alcohol consumption are associated with higher DFI levels [20]. These findings represent significant strides toward understanding male infertility and developing new approaches for diagnosing and treating fertility issues. Recent studies examined the genetic and environmental influences on the DFI and their impact on male infertility [45,46]. Their findings underscore the importance of identifying genetic factors affecting the DFI and their interaction with environmental factors such as age and dietary habits. Additionally, they found that genetic mutations can contribute to increased sperm DNA fragmentation, thereby affecting male fertility. Ogawa et al. demonstrated that a balanced diet containing micronutrient antioxidants can reduce oxidative stress and the DFI and consequently improve sperm function and the outcomes of in vitro fertilization (IVF)/Intracytoplasmic Sperm Injection (ICSI)–Embryo Transfer (ET) cycles [47].

Combining the findings from the studies included in this review, several conclusions can be drawn about the effect of the sperm DNA fragmentation index on embryo development, pregnancy outcomes, and assisted reproduction technology outcomes. Regarding the dynamics of embryonic development, it is found that lower levels of the DFI in sperm were associated with faster fetal morphokinetic parameters after ICSI. More specifically, the low-DFI group reached the blastocyst stage faster, and the study results suggest that lowering the DFI may positively affect embryonic development and may be predictive of pregnancy outcomes [36]. As far as implantation and pregnancy rates are concerned, high DFI levels negatively impact pregnancy rates after the implementation of assisted reproduction techniques. In the cohort study by Zhang et al., higher values of the DFI were associated with statistically significant lower pregnancy rates after IVF and ICSI cycles [44]. Similarly, in another study, the use of semen with lower DFI levels in ICSI cycles resulted in higher clinical pregnancy rates, lower miscarriage rates, and higher live birth rates compared to high-DFI semen, and therefore, the selection of low-DFI sperm results in better assisted reproductive technology (ART) outcomes [10].

Spermatogenesis is related to different cellular procedures in which a high number of genes are involved [48,49,50]. Different studies in humans revealed that genetic variants are closely related to spermatogenesis disorders. Such variants may be detected in endocrine-related genes (Gonadotropin-Releasing Hormone—GnRH, Follicle-Stimulating Hormone—FSH, Luteinizing Hormone—LH, Follicle-Stimulating Hormone Receptor—FSHR, Luteinizing Hormone Receptor—LHR), gonadal development-related genes (Azoospermia Factors—AZF, Wilms tumor gene 1—WT1, PR/SET domain 1—PRDM1, Stoltzfus blood group—SF), and meiosis-related genes (mutL homolog 1—MLH1, interferon regulatory factor 1—IRF1, PR/SET domain 9—PRDM9, SPO11 initiator of meiotic double strand breaks—SPO11), suggesting a possible interaction between reproductive-related genes and the DFI [51,52,53,54,55]. Additionally, it has been shown from numerous experiments that the expression of MicroRNAs (miRNAs) plays an indispensable role in spermatogenesis, affecting male infertility and the DFI [56,57,58]. Moreover, it was reported that interferon regulatory factor 1 (IRF1), a member of the interferon regulatory factor (IRF) family, is directly targeted by miR-383, which, by regulating interferon in cell apoptosis and the cell cycle, is involved in testicular spermatogenesis and the DFI [59]. In another study, the role of miRNAs in infertile males has been explored in different groups of fertile and infertile men, and the results indicate that the expression of miR-34c in the moderate oligoasthenoteratozoospermic and non-obstructive azoospermia groups was significantly elevated, correlating the aforementioned miRNA with the DFI [60]. Last but not least is the role of protamines, arginine-rich nuclear proteins that play a crucial role in the compaction of DNA, particularly in sperm cells during spermatogenesis. Protamines replace histones during the final stages of spermatogenesis, enabling a more condensed and stable chromatin structure that protects the genetic material from damage [61]. However, insufficient protamination or improper protamine−DNA binding can lead to incomplete chromatin condensation, making the DNA more vulnerable to oxidative stress and can result in increased DNA fragmentation [62,63,64].

Furthermore, regarding the correlation of the DFI with sperm parameters and quality, there is a clear negative correlation. Higher DFI levels were associated with lower motility [28]. Moreover, there are data that suggest a connection between body mass index (BMI) and sperm quality. A higher BMI has been associated with lower sperm count and motility by several studies. The fertilization rate is also declining with higher body mass index [65]. Abnormal sperm morphology has been also linked with obesity, leading to reduced semen ability to penetrate the ovum [66,67]. Oxidative stress is associated with DNA fragmentation and sperm cell damage [65]. Lastly, reduced progressive motility is also associated with higher DFI values and lower sperm mitochondrial concentration [68]. The same negative impact of increased DFI levels has also been observed in infertile males with increased static redox potential (sORP). More specifically, higher levels of sORP have a positive correlation with immotility percentage and a negative correlation with total motility [69].

A major limitation in the extrapolation of the results included and the universal utilization of standardized DFI values is the wide variety of techniques used for estimating the DFI. According to the WHO, the laboratory techniques for measuring the DFI are as follows: the TUNEL assay (terminal deoxynucleotidyl transferase (dUTP), nick end labeling, the single-cell gel electrophoresis assay (Comet Assay), the SCD assay (sperm chromatin dispersion test), and SCSA (acridine orange flow cytometry). The TUNEL assay involves the incorporation of biotinylated dUTP at 3′ ends of DNA strand breaks and may detect single- and double-strand breaks; the labeled bases can be quantified by using a fluorescent microscope or flow cytometry [70]. TUNEL is a sensitive and reliable technique, with minimal inter-observer variability, but requires expensive equipment and personnel training [71]. Consistent with the results of Agarwal et al., the TUNEL assay seems to be the most commonly utilized [72]. The Comet assay is an electrophoretic technique using the principle that smaller fragmented DNA will migrate faster than intact DNA [73]. The Comet assay is very sensitive, as it may be performed in very low sperm counts. It is also reproducible but demands an experienced observer [74]. The SCD assay is an indirect method for measuring SDF, as it depends on the susceptibility of chromatin to denaturation after the application of an acid treatment [75]. It is a simple assay which does not require the use of fluorescence and can evaluate subpopulations of degraded spermatozoa [71], but it has been criticized for high inter-observer variability [74]. Lastly, SCSA is a well-described and commonly utilized test. This technique uses acridine orange staining, which by binding to denatured or intact DNA will generate different fluorescence signals. The analysis is made using a flow cytometer, allowing for the simultaneous examination of a large number of cells. It is considered the most statistically robust and reproducible method but requires expensive equipment and personnel training [71].

## 6. Conclusions

In conclusion, the studies included in this review highlight collectively the importance of measuring the sperm DNA fragmentation index (DFI) in the assessment of male infertility, sperm quality, natural conception outcomes, and assisted reproductive technologies (ARTs). Sperm DNA fragmentation appears to influence pregnancy outcomes and the success rates of fertilization. Research investigating sperm DNA fragmentation in relation to recurrent pregnancy loss consistently shows its association with increased miscarriage probabilities. Moreover, studies examining sperm DNA fragmentation prior to fertility treatments such as IUI and IVF demonstrate links with higher pregnancy rates and live birth percentages. Nevertheless, the overall impact of sperm DNA fragmentation on pregnancy outcomes and male infertility in general requires further investigation. Lower levels of the DFI are associated with improved development of healthy embryos and higher pregnancy rates, particularly in ICSI cycles. Understanding the impact of the DFI on sperm functionality and its predictive value for outcomes in both assisted and natural reproduction could optimize fertility treatments. Longitudinal studies are necessary to evaluate changes in DFI levels over time, considering factors such as age, lifestyle, environmental exposures, and medical interventions. They would provide insights into the natural progression of the DFI and its impact on fertility outcomes.

## 7. Future Directions

Additionally, longitudinal studies are proposed to explore the effectiveness of interventions aimed at reducing DFI levels, such as changes in daily habits and lifestyle (diet, exercise, stress reduction), antioxidant supplements, and therapies. Various antioxidants such as vitamin C, vitamin E, Glutathione, Coenzyme Q10, and polyphenols, have been studied in the context of DNA fragmentation. Each of these antioxidants aims to mitigate oxidative damage and prevent DNA fragmentation through various mechanisms, and an increasing number of studies provide compelling evidence regarding their potential benefits for male infertility. Assessing the effects of these interventions on fertility outcomes could aid in developing targeted therapeutic strategies. Furthermore, developing new diagnostic techniques for assessing sperm DNA fragmentation, including advanced imaging techniques, biomarker tests, and genomic approaches, would constitute an additional proposal for future research in the field. These techniques could offer greater sensitivity compared to traditional DFI measurement tests. Moreover, exploring the utility of combining the DFI with other sperm biomarkers, such as sperm DNA integrity tests, mitochondrial function tests, and RNA tests, could enhance the accuracy of predicting fertility outcomes.

## Figures and Tables

**Table 1:** Included studies regarding association of DNA fragmentation and seminal parameters.

Study	Study Type	Sample	Outcome
Boushaba and Belaaoui (2015) [19]	Cohort study	26 infertile men	No significant correlation of DFI with sperm morphology and volume was found.
Xie et al. (2018) [18]	Case–control study	80 infertile men and 20 fertile men	The DFI showed no correlation between conventional semen parameters
Hosseinifar et al. (2015) [31]	Cohort study	20 men	The mean DFI was significantly higher in patients with teratozoospermia, compared to the control group (*p* < 0.001).
Lu et al. (2018) [25]	Cohort study	1010 infertile men	- A correlation of the DFI with decreased semen volume and a higher percentage of abnormal heads was found (*p* < 0.001). - A negative correlation between obesity, BMI, serum lipid, serum testosterone and seminal plasma levels, sperm concentration, and motility was found.
Vinnakota et al. (2019) [28]	Cohort study	1082 infertile men and 234 sperm donors	Men with a high DFI were older and had lower sperm motility than those with a normal DFI.
Le et al. (2019) [27]	Case–control study	318 infertile men	- The DFI showed significant correlations with abnormal head morphology and progressive motility, with a positive correlation with abnormal heads (r = 0.202, *p* = 0.0003) and a negative correlation with progressive motility (r = −0.168, *p* = 0.0027). - The DFI was associated with male age, smoking, and alcohol consumption (*p* < 0.05).
Jakubik-Uljasz et al.(2020) [30]	Cross-sectional study	523 men with teratozoospermia	A higher proportion of individuals with teratozoospermia had high SDF levels (>30%) and a higher odds ratio for high SDF levels compared to those without.
Zhang et al. (2021) [24]	Cohort study	2760 infertile men and 2354 men with women with unexplained miscarriage	- Sperm volume and total sperm count were statistically significantly lower in patients with a DFI > 30% compared to the control group. - Sperm concentration was statistically significant lower in patients with a DFI > 20% and a DFI > 30%
Green et al. (2020) [23]	Cohort study	234 couples	Men with a DFI >15% had significantly lower total motile sperm and sperm concentration compared to those with a DFI ≤ 15%.
Ferrigno et al. (2021) [29]	Cohort study	125 infertile men	Spermatozoa with abnormal morphology were more likely to have DNA damage (*p* < 0.001).
Antonouli et al. (2019) [22]	Clinical trial	150 couples	- A positive correlation between the DFI and advanced male age was found (r = 0.23, *p* < 0.05). - A negative correlation between total semen and motility was found (r = −0.29, r = −0.27; *p* < 0.05).
Wang et al. (2022) [21]	Clinical trial	381 couples	The DFI showed a negative correlation with sperm motility (r = −0.640, *p* < 0.01), sperm concentration (r = −0.289, *p* < 0.01), and the fertilization rate of IVF cycles.
Zhou et al. (2023) [26]	Cross-sectional study	93 couples	Logistic regression analysis indicated that the DFI had a negative correlation with asthenospermia (r = −0.37, *p* < 0.01).
Akhavizadegan et al. (2023) [20]	Cohort study	172 couples	Patients with abnormal semen analysis had significantly higher DFI levels compared to patients with normal or slightly abnormal semen analysis.

DFI: DNA fragmentation index, BMI: body mass index, SDF: sperm DNA fragmentation, and IVF: in vitro fertilization.

**Table 2:** Included studies regarding impact of DNA fragmentation on embryo kinetics, quality, and assisted reproduction outcomes.

Study	Study Type	Sample	Outcome
Wang et al. (2022) [21]	Clinical trial	381 couples	- The fertilization rate was significantly lower in the high (≥25%)-DFI group compared with the low (<25%)-DFI group using IVF (73.3% ± 23.9% vs. 53.2% ± 33.6%, respectively; *p* < 0.01). - The fertilization rate was equivalent in high- and low-DFI groups using ICSI.
Wdowiak et al. (2015) [36]	Clinical trial	165 couples who underwent ICSI	Lower DFI levels were associated with faster embryo development at the blastocyst stage.
Esteves et al. (2015) [10]	Cohort study	147 couples who underwent IVF-ICSI gave 77 testicular sperm samples (with lower DFI) and 87 ejaculated samples (with high DFI)	The clinical pregnancy rate was 51.9% using testicular samples versus 40.2% using ejaculated samples (*p* = 0.131); similarly, the miscarriage rate was 10.0% versus 34.3% (*p* = 0.012) and the live birth rate was 46.7% versus 26.4% (*p* = 0.007).
Zhang et al. (2016) [44]	Cross-sectional study	1316 couples in IVF and 266 couples in ICSI	The corresponding odds ratio (OR) of pregnant versus not pregnant was 10%.The DFI increase was 0.849 (95% CI, 0.738–0.976, *p* = 0.022) and 0.707 (95% CI, 0.559–0.893, *p* = 0.004) in the IVF andICSI programs, respectively.
Tello-Mora et al. (2018) [35]	Cross-sectional study	69 couples	The DFI was inversely correlated with viable blastocyst rates.
Omrani et al. (2018) [43]	Cross-sectional study	94 infertile men	- No significant difference in ICSI success rates was found between the low- (*p* = 0.597) and moderate (*p* = 0.235)-DFI groups. - In the high-DFI group, no pregnancy occurred.
Sun et al. (2018) [34]	Cohort study	390 couples	No significant differences in fertilization rate, euploid embryos, or pregnancy rate between the high- (≥30%) and low (<30%)-DFI groups after IVF or ICSI were found.
Siddhartha et al. (2019) [42]	Cohort study	105 infertile men	The pregnancy rate using ICSI was significantly lower in the positive-DFI group, compared to the group with negative DFI values (16.7% vs. 47.4%, *p* = 0.046).
Voncina et al. (2021) [41]	Cohort study	2713 infertile couples	- IVF with a normal DFI had a higher cumulative live birth rate compared to those with a high DFI (>20%). - A high DFI predicts a statistically significantly lower CLBR if IVF and not ICSI is applied in the first cycle.
Agsari et al. (2021) [33]	Clinical trial	42 couples	The higher DFI group showed a significant (*p* = 0.04) increase in the number of aneuploid embryos compared to the low-DFI group.
Wang et al. (2023) [32]	Case–control study	176 men	A negative correlation between the DFI and number of good quality embryos (rs = −0.347, *p* < 0.001) and live birth rate (rs = −0.185, *p* = 0.028) was found.
Bibi et al. (2022) [39]	Cohort study	700 couples	Sperm chromatin structural anomalies are significantly associated with a decreased fertilization rate (*p* = 0.009) and live birth rate (*p* = 0.006).
AmirJannati et al. (2024) [38]	Case–control study	870 couples	No significant differences in the fertilization rate, number, and quality of embryos were found.
Dar et al. (2013) [37]	Cohort study	150 men	No significant differences in fertilization and clinical pregnancy rates were observed.
Rex et al. (2021) [40]	Cohort study	357 couples	- The pregnancy rate was 9.9% when the initial DFI > 10 and increased to 21.7% when the initial DFI ≤ 10 (*p* < 0.005). - The live birth rate was 5% for an initial DFI > 10 and 14.2% for an initial DFI ≤ 10 (*p* < 0.005).

DFI: DNA fragmentation index, IVF: in vitro fertilization, ICSI: Intracytoplasmic Sperm Injection, and CLBR: cumulative live birth rate.

## Data Availability

Not applicable.
